# Bioactive metal-protein matrix for promoting MRSA infection wound therapy through bioenergy-induced angiogenesis

**DOI:** 10.7150/thno.112678

**Published:** 2025-06-09

**Authors:** Sihua Li, Junping Ma, Liuyang Zhang, Xiaoyan Qu, Long Zhang, Qian Huang, Bo Lei

**Affiliations:** 1 Key Laboratory of Shaanxi Province for Craniofacial Precision Medicine Research, College of Stomatology, Xi'an Jiaotong University, Xi'an 710000, China.; 2 Frontier Institute of Science and Technology, Xi'an Jiaotong University, Xi'an 710000, China.; 3 Department of Respiratory and Critical Care Medicine, The Second Affiliated Hospital of Xi'an Jiaotong University, Xi'an, 710004, China.

**Keywords:** bioactive materials, cellular metabolic state, MDRB wound repair, tissue engineering, angiogenesis

## Abstract

**Background:** Wound healing impaired by multidrug-resistant bacteria (MDRB) remains a significant clinical challenge, primarily due to persistent bacterial infection, excessive inflammation, overproduction of reactive oxygen species (ROS), and compromised vascularization. Importantly, the cellular metabolic state plays a vital role in regulating cellular behavior, and strategies aimed at enhancing cellular energy metabolism hold great promise for promoting tissue regeneration.

**Methods:** Herein, we present a multifunctional and bioactive silk fibroin-poly(citrate-curcumin)-metal-based biomimetic matrix (SFPC) designed to treat methicillin-resistant staphylococcus aureus (MRSA)-infected wounds by promoting bioenergy-induced angiogenesis.

**Results:** SFPC exhibited robust broad-spectrum antimicrobial, anti-inflammatory, intracellular ROS-scavenging, and pro-angiogenic properties. Notably, SFPC effectively enhanced mitochondrial membrane potential and promoted adenosine triphosphate (ATP) production in HUVECs, thereby accelerating angiogenesis through the controlled release of citrate.

**Conclusions:** This study suggests that SFPC is a promising alternative for the treatment of MRSA infected wounds and provides a facile approach for the development of a multifunctional hydrogel that promotes the healing of MRSA infected wounds at the level of cellular energy biology.

## Introduction

Multidrug-resistant bacteria (MDRB) infections in wound repair are among the most significant global challenges today, placing a tremendous burden on both patients and healthcare systems 1-2. Resistant bacteria colonize the wound site and often form a biofilm, which acts as a barrier, hindering the effectiveness of dressings and impeding immune system activation, thereby further delaying the healing process 3. Additionally, the overproduction of reactive oxygen species (ROS) caused by bacterial infection leads to lipid peroxidation, damaging DNA, proteins, and essential cellular components. This exacerbates the inflammatory response, impairs wound re-epithelialization, and ultimately contributes to wound deterioration and delayed healing 4-5. Another critical challenge in wound healing is impaired angiogenesis. Insufficient vascularization hampers the delivery of oxygen and nutrients while also limiting the removal of metabolic waste products. This disruption contributes to metabolic imbalances and endothelial dysfunction, ultimately leading to delayed or impaired wound healing. 6-8. In response to the challenges posed by MDRB infections, a growing number of wound dressings have been designed with multifunctional capabilities, including antimicrobial action, ROS scavenging, and the enhancement of angiogenesis 9-11. Although numerous strategies have shown encouraging results in the treatment of MDRB-infected wounds, it is often overlooked that pathological conditions can disrupt cellular energy metabolism. Such metabolic disturbances hinder tissue repair and regeneration, ultimately impeding wound healing.

The state of cellular energy metabolism is closely linked to the processes of tissue repair and regeneration 12. Elevated cellular energy metabolism facilitates mitosis and the swift production of anabolic substrates, ultimately promoting more efficient tissue repair and regeneration 13. Adenosine triphosphate (ATP) serves as the primary energy currency of the cell, supporting and sustaining all essential cellular activities 14. Sufficient energy availability is critical for successful wound repair. Continuous ATP synthesis is necessary to fuel key biological processes, including protein and lipid biosynthesis, signal transduction, mitosis, and cellular migration 15. Thus, modulating energy metabolism to accelerate tissue repair presents considerable promise for clinical application. Emerging bioenergetically active materials have been designed to integrate into the tricarboxylic acid (TCA) cycle through the formation of an intramitochondrial metabolic bypass, thereby enhancing mitochondrial membrane potential and ATP production, and ultimately promoting efficient bone tissue regeneration 13. However, there is a notable lack of biomaterials designed to modulate energy metabolism in the context of skin wound repair and regeneration.

Citric acid (CA), a key intermediate in the tricarboxylic acid (TCA) cycle, possesses a unique tricarboxylic structure that can be readily functionalized and modified. It plays an important role in biochemical metabolism and also exhibits anti-inflammatory and antioxidant properties 16. Therefore, incorporating citric acid into biomaterials for the treatment of drug-resistant bacterial infections in wound healing presents a promising strategy. Poly (octamethylene citrate) (POC), a biologically active polymer synthesized by melt polymerization of CA and diols, is one of the few polymeric biomaterials with significant biomedical potential. Its advantages include ease of synthesis, structural versatility, biocompatibility, biomimetic viscoelasticity, and the presence of modifiable functional groups 17. However, traditional POC is not water-soluble, its mechanical properties, bioactivity, and functionality are relatively uniform, limiting its ability to meet the diverse and complex demands of clinical applications 18. In recent years, our group has developed and synthesized PCE (poly(citrate)-ε-poly lysine), PCS (poly(citrate-glycol-siloxane)), and POCG-PEI (poly(citrate)-polyethylene glycol-polyethylenimine) through the further functionalization and modification of POC. We have also demonstrated their broad range of applications in bone regeneration, tumor therapy, anti-infection, and wound healing 19-22. Curcumin (Cur), a natural active small molecule, offers a wide range of advantages, including strong anti-inflammatory, antioxidant, and antimicrobial activities, as well as unique fluorescent properties. Therefore, utilizing curcumin for the further functionalization of POC presents a highly promising strategy for the treatment of drug-resistant bacterial infections in wound repair 23. Our group has developed an amphiphilic multifunctional polymer, poly (citric acid-polyethylene glycol-curcumin) (PCGC), which combines sustained curcumin release, photoluminescence, excellent biocompatibility, and strong anti-inflammatory and antioxidant properties. PCGC has demonstrated therapeutic efficacy in both acute lung injury and MRSA-infected wound repair, highlighting its promising biomedical potential. Significant progress has also been made in the development of poly (citric acid)-based hydrogels, which exhibit excellent mechanical properties, biocompatibility, and controllable degradability, making them highly versatile for a wide range of biomedical applications 24. These materials are capable of matching the mechanical properties of wounds, resisting bacterial infection, and reducing inflammation, demonstrating significant potential for wound repair. However, research on their role in regulating energy metabolism remains limited. Disrupted energy metabolism is a key factor contributing to the difficulty of healing infected wounds. Therefore, this study proposes a bioenergy enhancement strategy to improve the performance of poly (citric acid)-based hydrogels in wound repair by regulating energy metabolism.

In this study, we develop a multifunctional enzyme-crosslinked hydrogel, silk fibroin-poly (citric acid)-curcumin-Co^2+^ (SFPC), based on citrate-based polymers. In this system, the tyrosine residues of poly (citric acid)-curcumin (PCGC) and silk fibroin (SF) are crosslinked by horseradish peroxidase (HRP)/H_2_O_2_, and π-π physical crosslinking occurs between the benzene rings. Co^2+^ is chosen as the crosslinking agent due to its superior ability to enhance the hydrogel's mechanical properties, stability, and antibacterial performance, as well as its improved chemical stability and compatibility 25. The key to this strategy lies in our use of multiple interactions, including enzyme crosslinking, physical interactions, and ionic crosslinking, to prepare SFPC hydrogels with enhanced functionality and excellent mechanical properties. This approach facilitates their potential conversion into clinical applications, addressing the limitations of PCGC and SF, which suffer from poor mechanical properties, limited applications, and a narrow functional scope. In addition, the SFPC hydrogel integrates antibacterial, anti-inflammatory, antioxidant, and pro-vascularization functions at the macroscopic level, while also enhancing energy metabolism at the cellular level. These synergistic effects promote the repair and regeneration of MRSA-infected tissues. Subsequently, its physicochemical structure, multifunctional properties, and efficacy in MRSA-infected wound healing were thoroughly investigated.

## Materials and Methods

### Synthesis and characterizations of SFPC hydrogel

The raw silk was boiled in 0.01 M Na_2_CO_3_ for 30 min, then washed and dried. After that, it was dissolved in 9.3 M LiBr at 60 ℃ for 6 h, followed by dialysis (3500 MWCO) for 2 days. The dialyzed products were subsequently centrifuged three times at 1200 rpm for 20 min each at 4 °C. Finally, the centrifuged product was collected as SF after freeze-drying and stored at 4 °C for future use. The PCGC prepolymer was synthesized *via* a one-step melt polymerization reaction using CA (Sigma), polyethylene glycol (PEG, Energy), 1,8-octanediol (OD, Aladdin), and curcumin (Cur, Sigma) as raw materials. In brief, CA was added to a round-bottom flask and heated at 160 °C until it melted and became transparent. Then, OD and PEG (MW 1000 g/mol) were sequentially added and stirred. Subsequently, curcumin was introduced under a nitrogen atmosphere, and the temperature was lowered to 140 °C. The reaction was carried out under vacuum until the speed of the magnetic rotor fluctuated around 650 rpm. To purify the PCGC prepolymer, it was dissolved in deionized water and subjected to dialysis using a 3500 MWCO dialysis bag for 3 days. After dialysis, the PCGC prepolymer was freeze-dried and stored at 4 ℃ for future use. SFPC hydrogel is formed by catalytic oxidation crosslinking of phenol with HRP/H_2_O_2_ (Sigma), as well as chelation crosslinking with Co^2+^. In short, 250 mg PCGC prepolymer was dissolved completely in 500 μL deionized water (DI water) to prepare precursor solution A. Meanwhile, 80 mg SF with different masses (5, 10 and 15 mg) of Co^2+^ (cobalt nitrate hexahydrate) were added to 500 μL DI water to dissolve it completely (precursor solution B). Then, precursor solution A was added to solution B and thoroughly mixed. Afterwards, 340 U/mL of HRP and 0.01 M H_2_O_2_ were added and mixed well to obtain SFPC hydrogels with different doping levels of Co^2+^, which were completely crosslinked. The chemical structure of the SFPC hydrogel was characterized using ^1^H nuclear magnetic resonance (^1^H NMR) (Ascend 400 MHZ, Bruker) and Fourier transform infrared (FT-IR) spectroscopy (Alpha II, Bruker). The morphology and composition of the hydrogel were observed using scanning electron microscopy (SEM) (Quanta 250 FEG, FEI) and energy-dispersive X-ray spectroscopy (EDS). The rheological properties of the hydrogel were characterized by a TA rheometer (DHR-2).

### *In vitro* anti-bacterial activity, hemocompatibility and anti-oxidative activity investigation

The antibacterial ability of the hydrogel is crucial for preventing bacterial infection in the wound area. Therefore, this study investigated the broad-spectrum antibacterial activity of SFPC hydrogel against *Escherichia coli* (*E. coli*), *staphylococcus aureus* (*S. aureus*), and methicillin-resistant staphylococcus aureus (MRSA). The detailed process was described in the supporting information.

To evaluate the hemolytic properties of the hydrogel, red blood cells from Kunming mice were used. Fresh mouse blood was collected using the tail cutting method into a 1.5 mL eppendorf tube (EP tube) containing heparin. The sample was then centrifuged at 1000 rpm for 10 min and washed five times with PBS to obtain red blood cells. Hemolysis rate was calculated by the formula, Hemolysis ratio (%) = (A_Sample_ - A_PBS_)/(A_Triton_ - A_PBS_) × 100%. The detailed process was shown in the supporting information.

The DPPH (1,1-diphenyl-2-picryl-hydrazyl) (TCI) radical scavenging assay was used to evaluate the antioxidant capacity of SFPC hydrogels. In summary, hydrogels containing different concentrations of Co^2+^ ions doping, as well as a positive control (ascorbic acid (VC, Sigma)) and a negative control (DPPH), were placed at the bottom of 24-well plates. Then, 300 μL 1000 μM DPPH methanol solution was added to each group, followed by incubation at 37 °C for 30 min while being protected from light. Subsequently, 200 μL supernatant was transferred from each group to a new 2 mL centrifuge tube and diluted with methanol solution. The sample solutions were then photographed and recorded using a camera, and the absorbance values at 515 nm were measured by a UV spectrophotometer (Lambda 35, PerkinElmer) to calculate the anti-oxidation rate of the hydrogels. Anti-oxidation rate (%) = [1 - (A_DPPH_ - A_Sample_)/A_DPPH_] × 100%. In accordance, we further demonstrated the antioxidant function of SFPC hydrogels using the ABTS (Beyotime) free radical scavenging assay. Briefly, ABTS (negative control), SFP, SFPC and Trolox (positive control) were placed in a centrifuge tube along with 1 mL ABTS and co-cultivated for 15 minutes at 37 ℃ on a shaker protected from light. Subsequently, pictures were taken with a camera. Subsequently, the absorbance value at 734 nm was measured using UV-Vis spectroscopy to calculate the antioxidant rate.

### Cytocompatibility evaluation, intracellular ROS scavenging and anti-inflammatory evaluation

To test the cytocompatibility of the hydrogels, we determined the cytocompatibility of the hydrogels in L929 cells, HUVEC cells and RAW 264.7 cells, respectively, using the Alamar Blue^®^ kit (Invitrogen). The L929 cells and HUVECs, purchased from American Type Culture Collection. Briefly, 5000 L929 cells, HUVEC cells and RAW 264.7 cells were inoculated into 96-well plates, respectively. After cell apposition, the hydrogels were added and co-incubated with the cells for 24 h and 72 h, respectively. Subsequently, Alamar Blue^®^ was added and the absorbance at 560 nm/600 nm (excitation/emission) was measured by the microreader (SpectraMax Paragigm, Molecular Devices) after 4 h incubation with the cells to calculate the cell activity.

In addition, we used DCFH-DA (2',7'-Dichlorodihydrofluorescein diacetate) staining (Beyotime Biotechnology) to evaluate the ROS scavenging ability of the SFPC hydrogel. Briefly, sterile glass slides were placed in a 24-well plate, and a certain number of macrophages were seeded into the wells. After the cells adhered to the glass surface, they were induced to produce ROS by co-culturing with 400 ng/mL lipopolysaccharides (LPS, Sigma) for 12 h. After co-incubation with the material for 48 hours, the cells were stained with DCFH-DA for 40 min, and the fluorescence intensity was observed using confocal laser scanning microscope (CLSM, Olympus FV1200). Image J was used for quantitative analysis of fluorescence intensity.

To assess the anti-inflammatory ability of SFPC hydrogel *in vitro*, RAW 264.7 cells were seeded into 12-well plates. After 24 h induction by LPS (400 ng/mL), the hydrogels were added to the culture medium and co-cultured with the cells for 48 h. Total RNA was extracted using Trizol (Beyotime), and cDNA was synthesized using the First-Strand cDNA Synthesis Kit (G486, abm) following the manufacturer's instructions. The gene expression level of *TNF-α*, *IL-1β* were quantified by RT-qPCR (Quantitative Real-time polymerase chain reaction) (Applied Biosystems 7500), with *β-actin* serving as the housekeeping gene. The detailed process was shown in the supporting information.

### Vascular endothelial cells migration, tube formation and vascularization analysis

The migration of endothelial cells, tube formation, and vascularization are essential biological processes for skin tissue regeneration. The effect of SFPC hydrogel on endothelial cell migration was assessed using a scratch assay. Briefly, HUVEC cells were seeded at a density of 10^5^ cells in a 12-well plate. Once the cell confluence reached above 90%, a 10 μL sterile pipette tip was used to vertically scratch the cell monolayer, inducing a cell wound. The cell debris was washed off with PBS, and then different materials were added and co-incubated with the cells. Optical microscopy images were taken at 0 h and 36 h, and the scratch area was calculated using Image J to determine the migration rate.

100 μL matrix gel (BD Biosciences, US) was evenly spread on a 48-well plate, avoiding the formation of bubbles, and then incubated at 37 ℃ for 30 min to allow it to solidify into a gel. Subsequently, HUVEC cells were seeded on the matrix gel at a density of 10^5^ cells and co-incubated with SFPC hydrogel for 8 h. The cells were then stained with Calcein-AM, and fluorescent images were obtained using a fluorescence microscope (BX53, Olympus). Image J was used to analyze the tube formation indices, such as the number of branches and the number of isolated segments.

By detecting the expression of angiogenesis-related genes *VEGF*, *CD31*, and *HIF-1α*, the impact of SFPC hydrogel on angiogenesis was evaluated. In simple terms, HUVEC cells were seeded into a 12-well plate, and the hydrogel was added to the culture dish for co-culturing with the cells. After 48 h, the expression of angiogenesis-related genes *VEGF*, *CD31*, and *HIF-1α* was detected by RT-qPCR. The detailed process was shown in the supporting information.

### Evaluation of cellular energy metabolism

Energy metabolism has a major role in tissue repair and regeneration. Adenosine triphosphate (ATP) is a primary source of cellular energy and is critical in numerous physiological and metabolic processes. Mitochondrial membrane potential (ΔΨm) is a direct chemical driver of ATP synthesis. The effect of SFPC hydrogels on ΔΨm was evaluated by JC-1 staining (Shanghai Life-iLab Biotech Co., Ltd). HUVEC cells were inoculated in 96-well plates and incubated with the material for 24 h. Where Carbonyl cyanide 3-chlorophenylhydrazone (CCCP) is the negative control group, indicating that the ΔΨm is completely disrupted. Subsequently, after staining with JC-1 for 15 min, the fluorescence intensity was measured with the microplate reader (red fluorescence: E_X_/E_m_=550 nm/600 nm; green fluorescence: E_X_/E_m_=485 nm/535 nm), and the ratio of red to green fluorescence was calculated to be the value of ΔΨm. At the same time the cells were stained with JC-1 and observed under a fluorescence microscope. Red fluorescence is the JC-1 aggregate form indicating enhanced membrane potential and elevated metabolism. On the contrary, green fluorescence is the form of JC-1 monomer, which indicates a decrease in membrane potential and a slowdown in metabolism. Flow cytometry was also applied to verify the effect of SFPC hydrogels on mitochondrial membrane potentials. The SFPC hydrogel-treated cells were collected and stained with JC-1, and then detected on a flow cytometer (CytoFLEX, Beckman Coulter). Among them, FITC channels are represented as JC-1 monomer form, indicating apoptosis. PE channels are represented as JC-1 aggregate form, indicating elevated membrane potential and enhanced metabolism. To further illustrate the effect of SFPC hydrogels on cellular energy metabolism, ATP production was examined. HUVEC cells were incubated with the material for 48h and then the cells were lysed using cell lysis solution. The intracellular ATP content was subsequently determined using an ATP colorimetric assay kit (Beijing Ita Biotechnology Co., Ltd) according to the supplier's instructions.

Cellular uptake of citric acid is a prerequisite for SFPC hydrogels to improve their metabolic status. To verify cellular uptake, PCGC-treated cells were observed on CLSM, where DAPI is indicated as the nucleus, and PCGC generates green fluorescence on its own under 488 nm laser. Subsequently, in order to detect the effect of citric acid entering the cells on the TCA cycle, we determined the intracellular citrate abundance. Specifically, after incubating the cells with the material for 24 h, the cells were lysed and the citric acid content of the cell lysates was assayed according to the instructions of the kit (Citrate Microplate Assay Kit, absin).

### *In vivo* MRSA-infected wound repair evaluation

All experimental procedures involving animals were carried out in accordance with institutional guidelines and approved by the Laboratory Animal Center of Xi'an Jiaotong University. Validation of SFPC hydrogel on wound repair by constructing a full thickness injury model in MRSA-infected Kunming mice (female, 8 weeks, 30-32 g). Specifically, the mice were anesthetized, their backs were shaved, and the dorsal skin was excised to form two circular wounds of approximately 7 mm. Subsequently, 10 μL bacterial suspension of MRSA (10^7^ CFU/mL) was added vertically dropwise to the wound area to induce infection, followed by treatment of the wounds with SFP hydrogel, SFPC hydrogel, and after that, uniformly covered with 3M Tegaderm film. On 3 d, 7 d, and 14 d, photographs of the wound were taken, and the wound area was quantified using Image J to assess the progress of wound healing. Meanwhile, to evaluate the *in vivo* antimicrobial performance of the hydrogel, the excised skin was shaken vigorously in PBS for 1 min to completely dislodge the bacteria from the wound. Finally, the bacterial suspension was inoculated onto LB agar plates to observe the colony growth. In addition, to further evaluate the growth of the wound, the excised skin was stained with H&E to observe epidermal healing and the growth of skin appendages such as hair follicles. Masson staining was used to evaluate collagen deposition in wounds on day 14. In order to verify the anti-inflammatory and vascularization properties of SFPC hydrogel, IL-6 immunofluorescent staining was performed on 3 d wounds and VEGF immunofluorescent staining was performed on 14 d wounds, and the tissue morphology as well as the positive expression were observed under the microscope.

### Statistical analysis

All results data are expressed as mean ± standard deviation. Statistical differences were determined using the student's t-test. Differences were considered statistically significant when the probability value (*p* value) was less than 0.05 (^*^p < 0.05, ^**^ *p* < 0.01). Origin 2018 software was used for statistical and analytical purposes.

## Results and Discussion

### Synthesis and characterizations of SFPC hydrogel

**Scheme 1:**
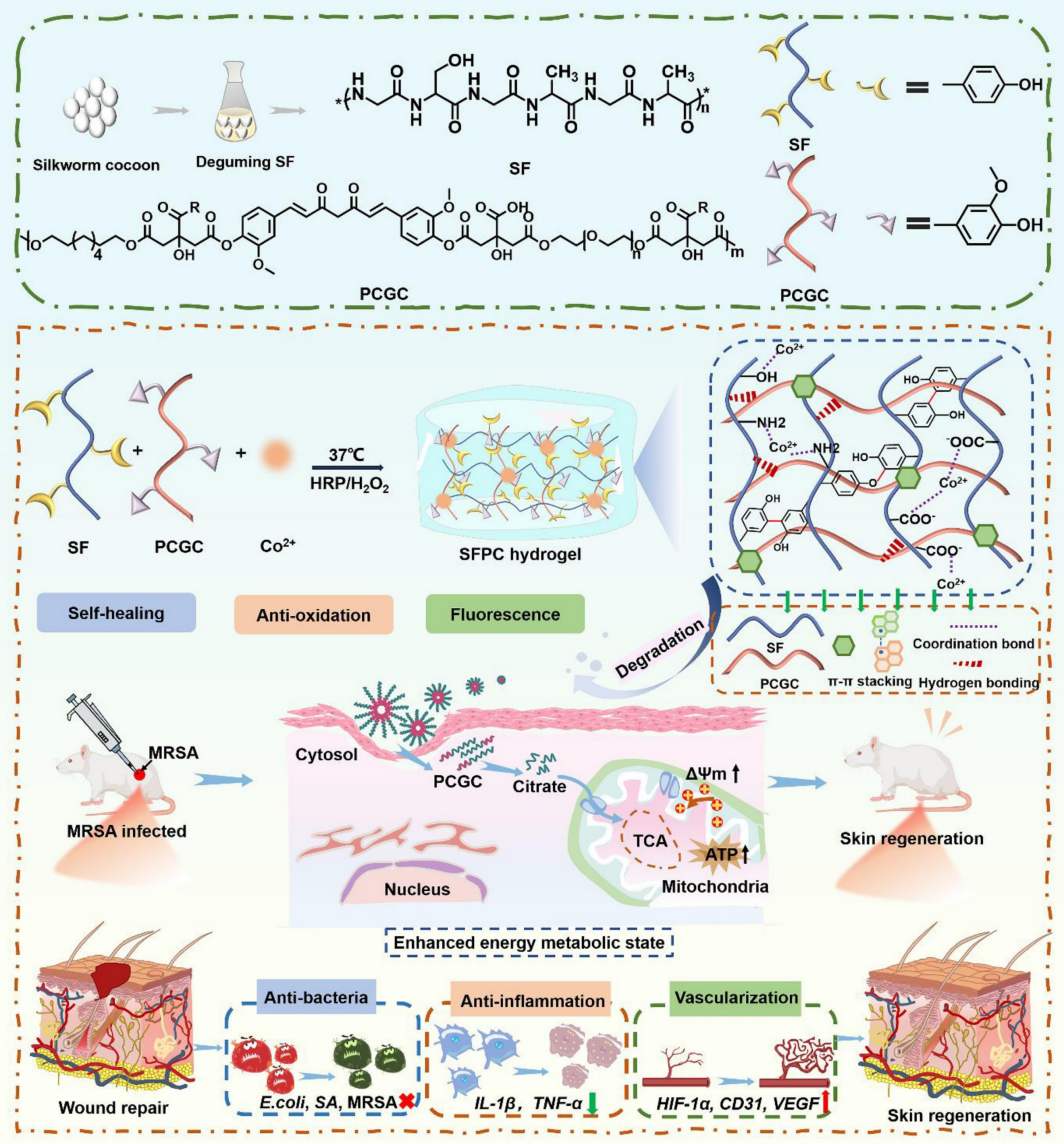
Schematic illustration of the synthesis of SFPC hydrogel with multifunctional properties for potential application in MRSA infected wound healing and skin regeneration.

**Figure 1:**
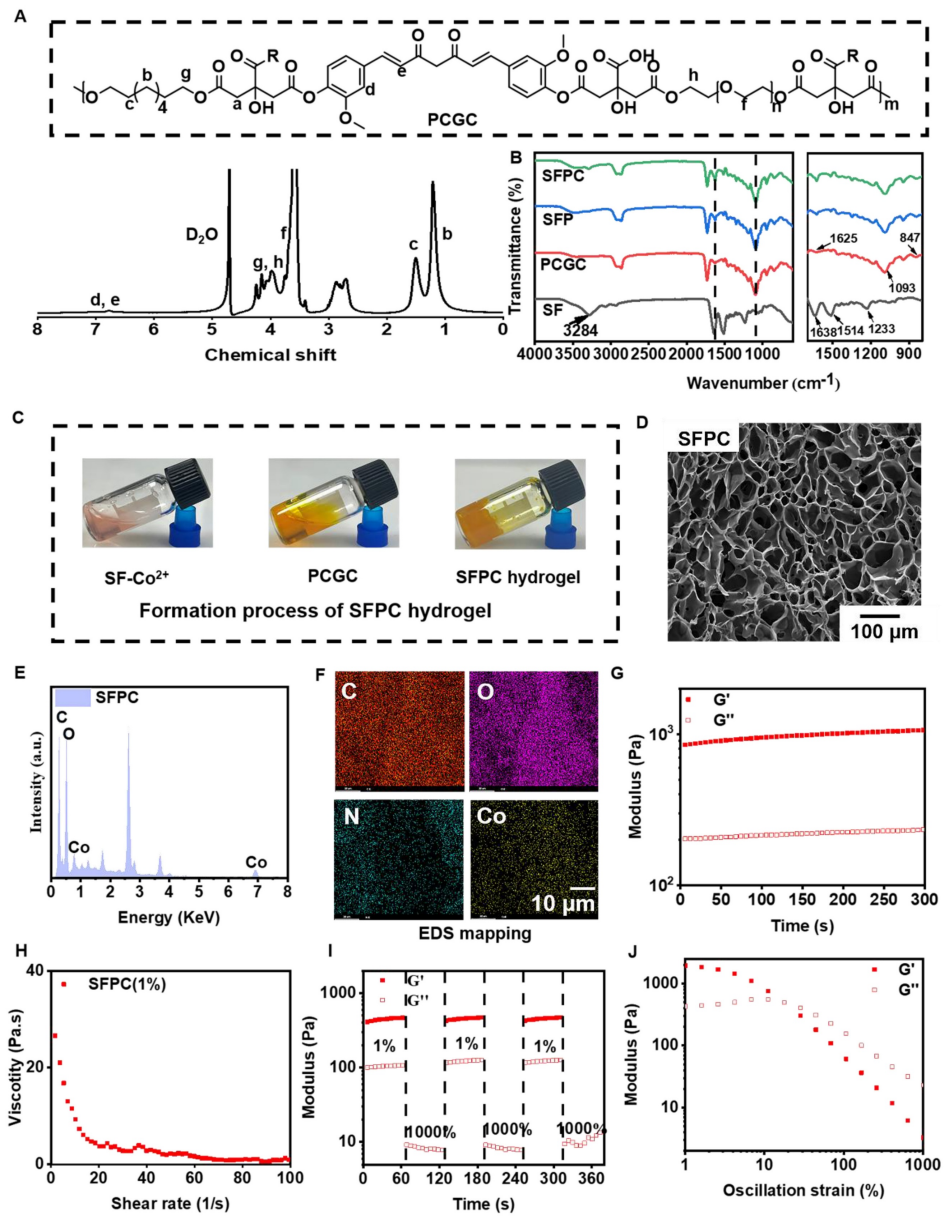
** Physical and chemical characterization of SFPC hydrogel.** (A) ^1^H NMR spectra of PCGC polymers. (B) FT-IR spectra of SF, PCGC, SFP and SFPC hydrogel. (C) Schematic diagram of the gel formation process of SFPC hydrogel**.** (D) Typical SEM images of SFPC hydrogels. (E) EDS spectra of the SFPC hydrogel. (F) Mapping images of each element in SFPC hydrogel (C, O, N and Co). (G) G′ and G″ of SFPC hydrogels. (H) Viscosity of SFPC hydrogel along with the shear rate from 1 1/s to 100 1/s. (I) G′ and G″ of SFPC hydrogel along with 2 times strain cycles between 1% and 1000%. (J) G′ and G″ of SFPC hydrogel along with the oscillation strain from 1% to 1000%.

**Scheme 1** illustrates the preparation, multifunctional physicochemical properties and biological characterization of SFPC hydrogels and their biomedical applications in the efficient treatment of MRSA infected wound repair. **Figure 1** demonstrates the characterizations of SFPC hydrogels. Poly (citrate-polyglycol-curcumin) PCGC was synthesized by a simple one-step melt polymerization. According to the ^1^H NMR of PCGC polymer, the solvent peaks of D_2_O were located at 4.7 ppm. The multiple peaks of methylene (-CH_2_-) in CA were located between 2.5 and 3.0 ppm. The multiple peaks of methylene (-CH_2_-) in OD were located at 1.2 ppm,1.5 ppm, 3.9 ppm and 4.1 ppm. In addition, the multiple peaks at approximately 7-8 ppm were attributed to the methylene (-CH_2_-) and the hydrogens on the phenyl ring of curcumin. And the methylene (-CH_2_-) characteristic peaks of PEG appear at 3.5, 4.2, and 4.3 ppm (**Figure 1A**). According to the FT-IR results, the peak at approximately 1638 cm^-1^, 1514 cm^-1^ and 1233 cm^-1^ were attributed to the amide I (-C=O-stretching), amide II (-C-N- stretching and -N-H- deformation), and amide III (-C-N- stretching and -N-H- deformation) of the SF, respectively. In the PCGC spectrum, the 1730 cm^-1^ peak arose from the ester carbonyl (C=O). The 2863 cm^-1^ peak corresponded to methylene (-CH_2_-) stretching. The 3435 cm^-1^ peak resulted from hydroxyl (-OH) stretching and unreacted carboxyl groups in hydrogen bonds. These peaks were also present in the SFP and SFPC spectra, confirming the prepolymer's role in hydrogel formation (**Figure 1B**). These results proved the successful synthesis of the SFPC hydrogel. **Figure 1C** demonstrates the gel formation process of SFPC hydrogel, the precursor SF-Co^2+^ solution A and the precursor PCGC solution B, both of which can form SFPC hydrogel after mixing and adding HRP/H_2_O_2_. The morphology and microstructure of the hydrogel were evaluated by SEM, and the SFP and SFPC hydrogel possessed an obvious three-dimensional porous structure. The pore size of SFPC hydrogels (40.34 μm) was slightly reduced compared to SFP hydrogels (74.15 μm), which was attributed to the doping of Co^2+^ increasing the crosslink density of the hydrogels (**Figures 1D and S1**). EDS was further used to evaluate the composition of the SFPC hydrogel (**Figure 1E**). The uniform distribution of C (red), O (purple), N (green) and Co (yellow) elements in the SFPC hydrogel can be significantly observed from the EDS mapping (**Figure 1F**). In addition, we evaluated the mechanical properties of SFPC hydrogels by the rheological testing. The storage modulus (G′) of SFPC hydrogels was significantly higher than their loss modulus (G″), which proved the successful formation of hydrogels (**Figure 1G**). As the shear rate gradually increased from 1 1/s to 100 1/s, the viscosity of SFPC hydrogel decreased significantly from 26.6 Pa**·**s to 0.96 Pa**·**s, which proved its good shear-thinning property (**Figure 1H**). The modulus of SFPC hydrogel did not change significantly after 3 cycles of high (1000%) and low (1%) oscillation strains, which proved that it recovered quickly with good self-healing ability (**Figure 1I**). In addition, **Figure 1J** shows the significant shear-thinning behavior of SFPC hydrogels.

### *In vitro* anti-bacterial activity, hemocompatibility and anti-oxidative activity investigation

**Figure 2:**
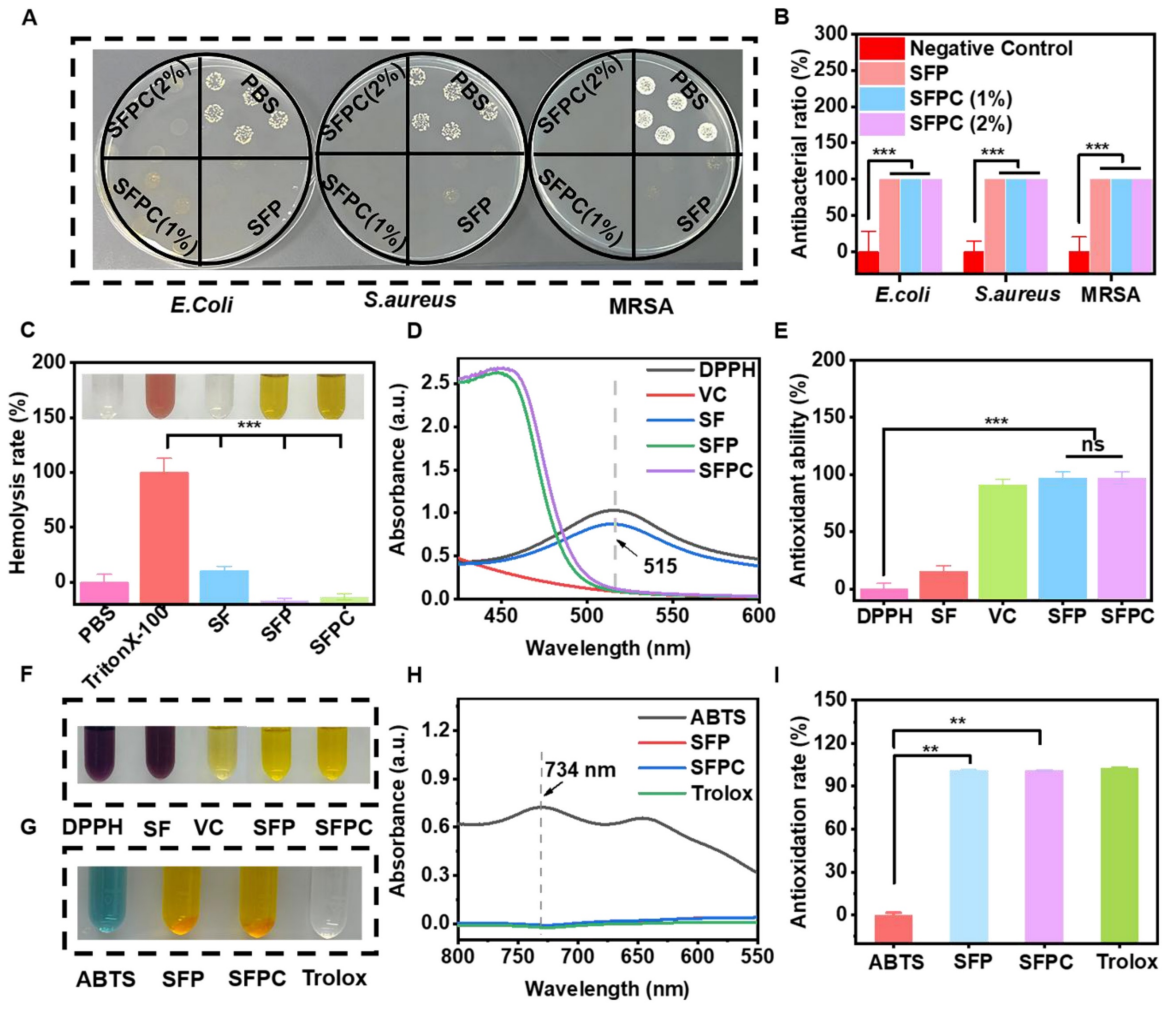
** Anti-bacterial activity, hemocompatibility and anti-oxidative activity assement and *in vitro*.** (A) Photographs of *E.coli*, *S.aureus* and MRSA colonies treated with different hydrogels and (B) corresponding statistics. (C) Hemolysis rate of red blood cells incubated with different samples at 37 ℃ for 1 h. (Illustration: Optical photo of mouse red blood cells after incubation of different samples for 1 h). (D) UV-Vis absorbance of DPPH, SF, SFP, SFPC hydrogels and VC at 515 nm after incubating with DPPH at 37 °C for 30 min. (E) Antioxidant capacity of various samples. (F) Hydrogel diagram after incubation with DPPH at 37 ℃. (G) Optical photo of different samples after incubation with ABTS. (H) UV-Vis absorbance of ABTS, SFP, SFPC hydrogels and Trolox at 734 nm after incubating with ABTS. (I) Antioxidation rate of various samples based on ABTS assay. ^***^p < 0.001, ^**^p < 0.01.

The antibacterial activity of SFPC hydrogels against *E. coli*, *S. aureus* and MRSA was also evaluated. After 2 hours of co-culture with the SFPC hydrogel, no colony growth was observed in SFP and SFPC groups. In contrast, the PBS group exhibited intensive colony growth (**Figure 2A**)**.** Quantitative analysis reveals a consistent trend, with SFPC hydrogels demonstrating nearly 99.9% antibacterial efficacy (**Figure 2B**). These results indicated that SFPC hydrogels exhibited excellent broad-spectrum antibacterial properties. Good biocompatibility is a prerequisite for the subsequent use of biomaterials. The hemocompatibility of SFPC hydrogels was evaluated through a hemolysis test. No hemolysis was observed in the SFPC hydrogel and negative control PBS groups, while significant hemolysis occurred in the positive control Triton X-100 group. (**Figure 2C**). The hemolysis rate was further quantified by measuring the absorbance at 540 nm. The results showed that the hemolysis rate of the SFPC group was less than 5%, demonstrating that SFPC hydrogels exhibit good hemocompatibility and are suitable for use in subsequent experiments.

The antioxidant capacity of SFPC hydrogels was assessed using the DPPH free radical scavenging assay. The results showed that the DPPH solution changed from its original purple color to yellow after incubation with the SFPC hydrogel, indicating its antioxidant activity (**Figure 2F**). The antioxidant activity of the hydrogel was calculated based on UV absorption data at 515 nm. The results showed that the antioxidant rate of the SFPC hydrogel reached 97.2%, which is comparable to that of vitamin C (91.0%) (**Figure 2D and figure 2E**). Additionally, the ABTS free radical scavenging assay demonstrated that the blue color of the solution faded in both the SFP and SFPC groups (**Figure 2G**). Additionally, the absorbance at 734 nm was significantly lower in the SFPC group compared to the ABTS group (**Figure 2H**). The quantitative data showed that the antioxidant rate of the SFP and SFPC groups was significantly higher than that of the ABTS group and comparable to that of the Trolox group (**Figure 2I**). The excellent antioxidant capacity of SFPC hydrogels stems from curcumin, which scavenges free radicals through its phenolic hydroxyl groups and β-diketone moiety, while also activating antioxidant pathways such as Nrf2-ARE and NF-κB to upregulate SOD and CAT. Unlike vitamin C, which rapidly oxidizes in aqueous environments, the three-dimensional network of SFPC hydrogels encapsulates curcumin, preventing degradation and allowing for sustained antioxidant release. This provides prolonged efficacy in wound healing, offering a distinct advantage over other materials 26.

### Cytocompatibility, intracellular ROS scavenging and anti-inflammatory evaluation

**Figure 3:**
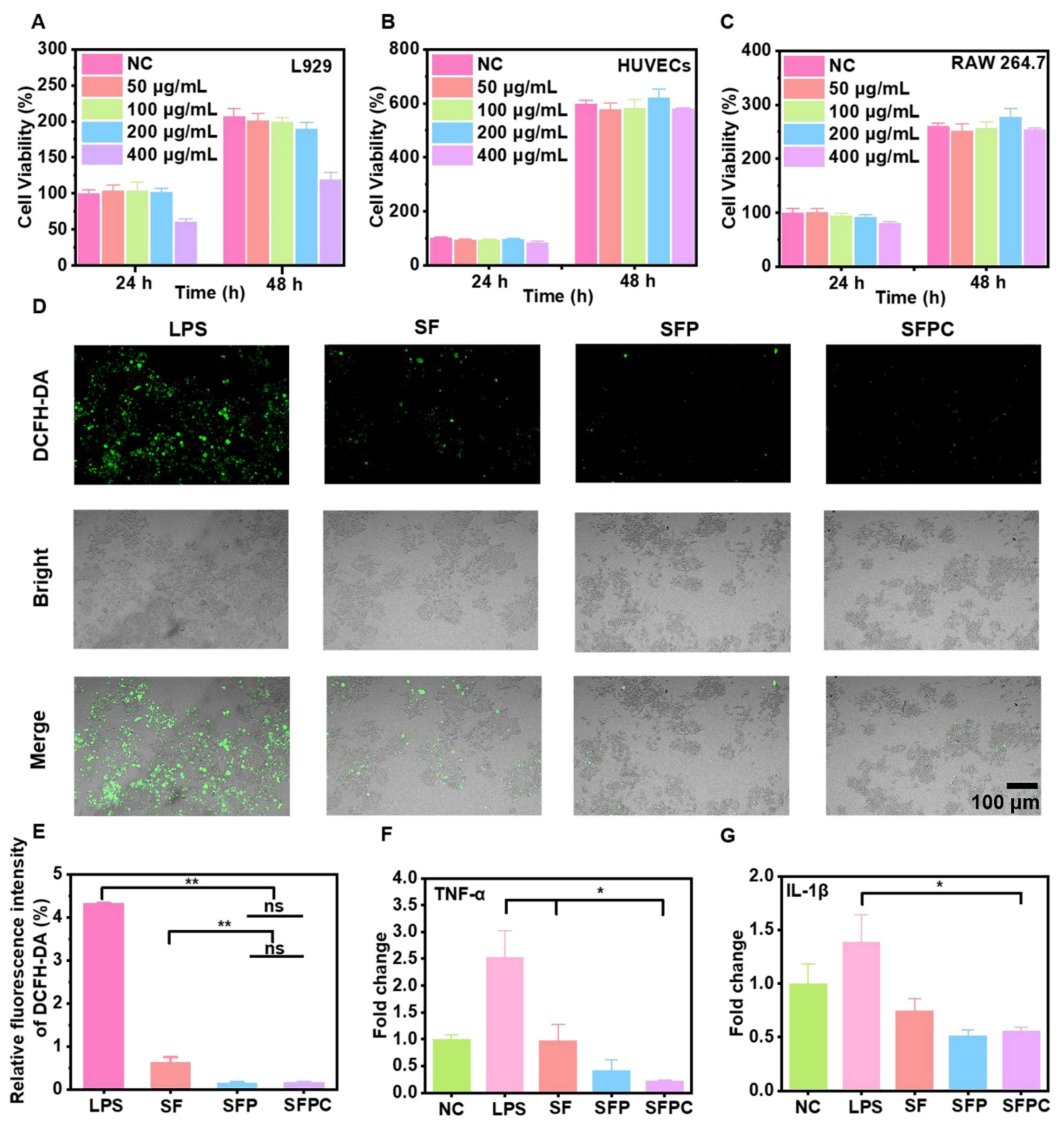
** Cell viability, intracellular ROS clearance and anti-inflammatory evaluation.** Cell viability of L929 cells (A), HUVEC cells (B) and RAW 264.7 cells (C) after treatment with different concentration of SFPC hydrogel. (D) CLSM image of cells stained by DCFH-DA after LPS treatment. (E) Relative fluorescence intensity of DCFH-DA. (F) *TNF-α* expression of LPS-treated RAW 264.7 cells incubated with SFPC hydrogel. (G) *IL-1β* expression of LPS-treated RAW 264.7 cells incubated with SFPC hydrogel. ^**^p < 0.01, ^*^p < 0.05, ns means not significant.

Cytocompatibility is a critical factor in evaluating biomaterials, and good cytocompatibility is essential for effective tissue repair and regeneration. The cytocompatibility of SFPC hydrogels was assessed using HUVECs, RAW 264.7 cells, and L929 cells. For L929 cells, a certain degree of cytotoxicity was observed at SFPC concentrations up to 400 μg/mL. However, for both HUVEC and RAW 264.7 cells, SFPC hydrogels exhibited good cell viability, even at concentrations as high as 400 μg/mL (**Figure 3A-C**). These results indicated that the SFPC hydrogel exhibited good cytocompatibility and was suitable for use in subsequent experiments.

The level of intracellular ROS is closely linked to cell proliferation and vascularization. Low levels of ROS promote wound healing by enhancing vascularization and cell migration, while elevated ROS levels impede healing by damaging essential intracellular lipids, DNA, and proteins, ultimately leading to cell death 27. Intracellular ROS levels were measured using DCFH-DA staining to evaluate the ROS scavenging ability of SFPC hydrogels. The LPS group exhibited a strong green fluorescence signal, indicating that LPS successfully induced a significant production of ROS. In contrast, green fluorescence was markedly reduced after SFPC treatment, which can be attributed to the excellent ROS scavenging properties of curcumin (**Figure 3D**). Furthermore, the quantitative statistics showed a similar trend, with the fluorescence intensity of the SFPC group being significantly smaller than that of the LPS group (**Figure 3E**). These results indicated that SFPC hydrogels possessed good ROS scavenging activity.

Good anti-inflammatory activity is essential for wound repair and regeneration 28. Biomaterials with anti-inflammatory activity promote wound healing by facilitating the transition from the inflammatory to the proliferative phase. The anti-inflammatory effect of SFPC hydrogels was evaluated by measuring the mRNA expression levels of inflammatory factors (*TNF-α* and *IL-1β*). After LPS induction, the inflammatory factors were significantly upregulated compared to the control group, confirming the successful induction of the inflammatory model. Following SFPC hydrogel treatment, the levels of these inflammatory factors were significantly downregulated (**Figure 3F and 3G**). Oxidative stress and inflammatory responses are mutually reinforcing, creating a vicious cycle. Excessive ROS can activate signaling pathways such as MAPK and NF-κB, leading to the release of pro-inflammatory cytokines like *TNF-α*, *IL-6* and *IL-1β*. As a central regulator of inflammation, NF-κB activation is enhanced by ROS-induced phosphorylation and nuclear translocation, promoting the expression of inflammatory genes. Additionally, ROS can further drive NF-κB nuclear translocation and cytokine release by activating the PI3K/Akt pathway 29. The anti-inflammatory mechanism of the SFPC hydrogel may be attributed to the effect of PCGC, particularly its ability to downregulate NF-κB expression, thereby inhibiting the inflammatory response. Moreover, PCGC effectively suppresses M1 macrophage polarization, further enhancing its anti-inflammatory effects 24. Furthermore, the ROS-scavenging effect of the SFPC hydrogel disrupts the ROS-inflammation feedback loop, promoting a pro-regenerative microenvironment. These results indicated that SFPC hydrogel exhibited excellent biocompatibility, along with strong anti-inflammatory and antioxidant activities. Therefore, SFPC hydrogel possessed great potential for application in the repair of MRSA-infected wounds.

### Vascular endothelial cell migration, tube formation and vascularization analysis

**Figure 4:**
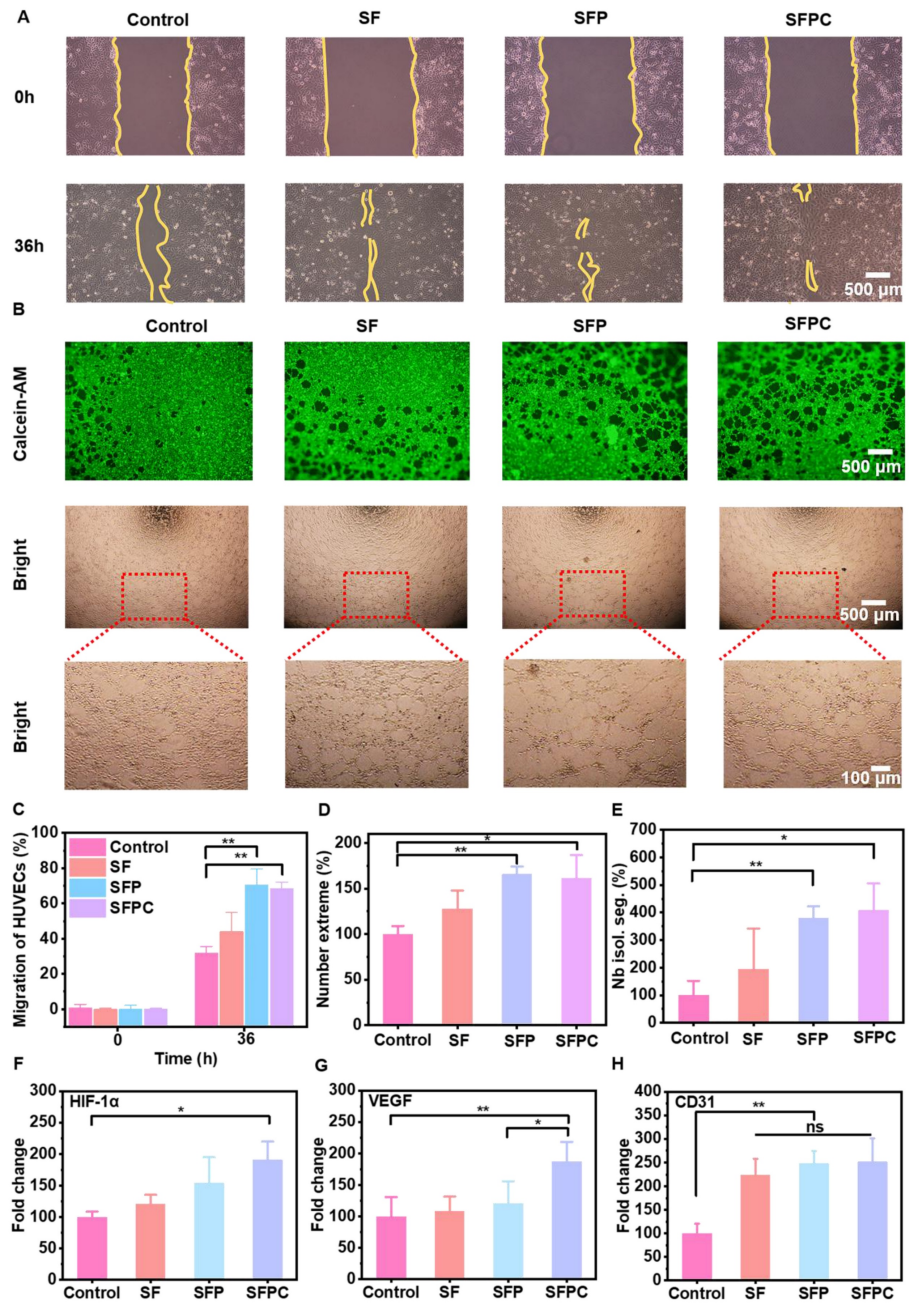
** Analysis of migration, tube formation and vascularization of vascular endothelial cells under different conditions.** (A) Migration images of HUVEC cells at 0 and 36 h. (B) Tube formation photographs. (C) Quantitative statistical results of mobility of HUVEC cells treated with SFPC hydrogel. Quantification of the number extreme (D) number isolation Segment (E) about tube formation. (F) *HIF-1α* expression of HUVEC cells incubated with SFPC hydrogel. (G) *VEGF* expression of HUVEC cells incubated with SFPC hydrogel. (H) *CD31* expression of HUVEC cells incubated with SFPC hydrogel. ^**^p < 0.01, ^*^p < 0.05, ns means not significant.

The migration and tube formation of HUVECs are critical for vascular regeneration and neovascularization 30. Blood vessels are essential for transporting nutrients, oxygen, and other vital substances to the wound area, providing the biological support necessary for wound healing and promoting tissue repair. The ability of SFPC hydrogels to enhance HUVEC cell migration was assessed using a scratch assay. As shown in **Figure 4A**, after 36 hours of incubation, the scratches in the SFPC group were nearly closed, whereas the control group still exhibited visible scratches. After 36 hours, the migration rate in the SFPC group reached 68.6%, while the migration rate in the control group was only 32.0% (**Figure 4C**). These results demonstrated that SFPC hydrogels have favorable ability to promote cell migration of HUVEC cells.

In addition, the tube formation ability of SFPC hydrogels was demonstrated by simulating vascularization on matrix gel. As shown in **Figure 4B**, the SFPC group formed a higher density of tubes with more branches and a more complex morphology. In contrast, the control group exhibited fewer tubes with a simpler morphology. Quantitative analysis of the number of extremes and isolated segments revealed that the SFPC group had significantly more extremes compared to the control group (**Figure 4D**). Meanwhile, the tube length in the SFPC group was more than four times greater than that in the control group (**Figure 4E**).

To explore the potential mechanism of SFPC hydrogel in promoting vascularization, we further investigated the expression of angiogenic genes (*HIF-1α*, *VEGF* and CD31). *HIF-1α*, a hypoxia-inducible factor, plays a key role in inducing local hypoxia, which in turn promotes the high expression of its downstream target gene *VEGF* 31. *VEGF*, a prominent marker of vascularization, promotes vascular endothelial cell proliferation, migration and tube formation 32. *CD31* represents a cell adhesion factor which is essential for neovasculogenesis by enhancing cell adhesion and signaling 33. To further elucidate the underlying mechanism by which SFPC hydrogels promote HUVEC cell migration and tube formation, we evaluated the mRNA expression levels of *HIF-1α*, *VEGF*, and *CD31* using RT-qPCR. The results indicated that *HIF-1α* expression was upregulated nearly twofold in SFPC-treated cells compared to the control group (**Figure 4F**). At the same time, the expression level of the downstream gene *VEGF* was also nearly 2-fold higher than that of the control group (**Figure 4G**). Moreover, the expression of *CD31* was significantly higher than that of the control group (**Figure 4H**). These results demonstrated that SFPC hydrogels can stabilize the expression of *HIF-1α* through Co^2+^ doping, thereby upregulating *VEGF* expression. Furthermore, SFPC, as a three-dimensional scaffold, mimicked the physical microenvironment of the extracellular matrix, enhancing HUVEC adhesion and motility. Through the synergistic actions of *VEGF* and *CD31*, SFPC hydrogels significantly promoted HUVEC migration, tube formation, and vascularization, showing promising potential for MRSA-infected wound healing. These findings further supported the potential of SFPC in promoting angiogenesis and tissue regeneration.

### Evaluation of cellular energy metabolism

**Figure 5:**
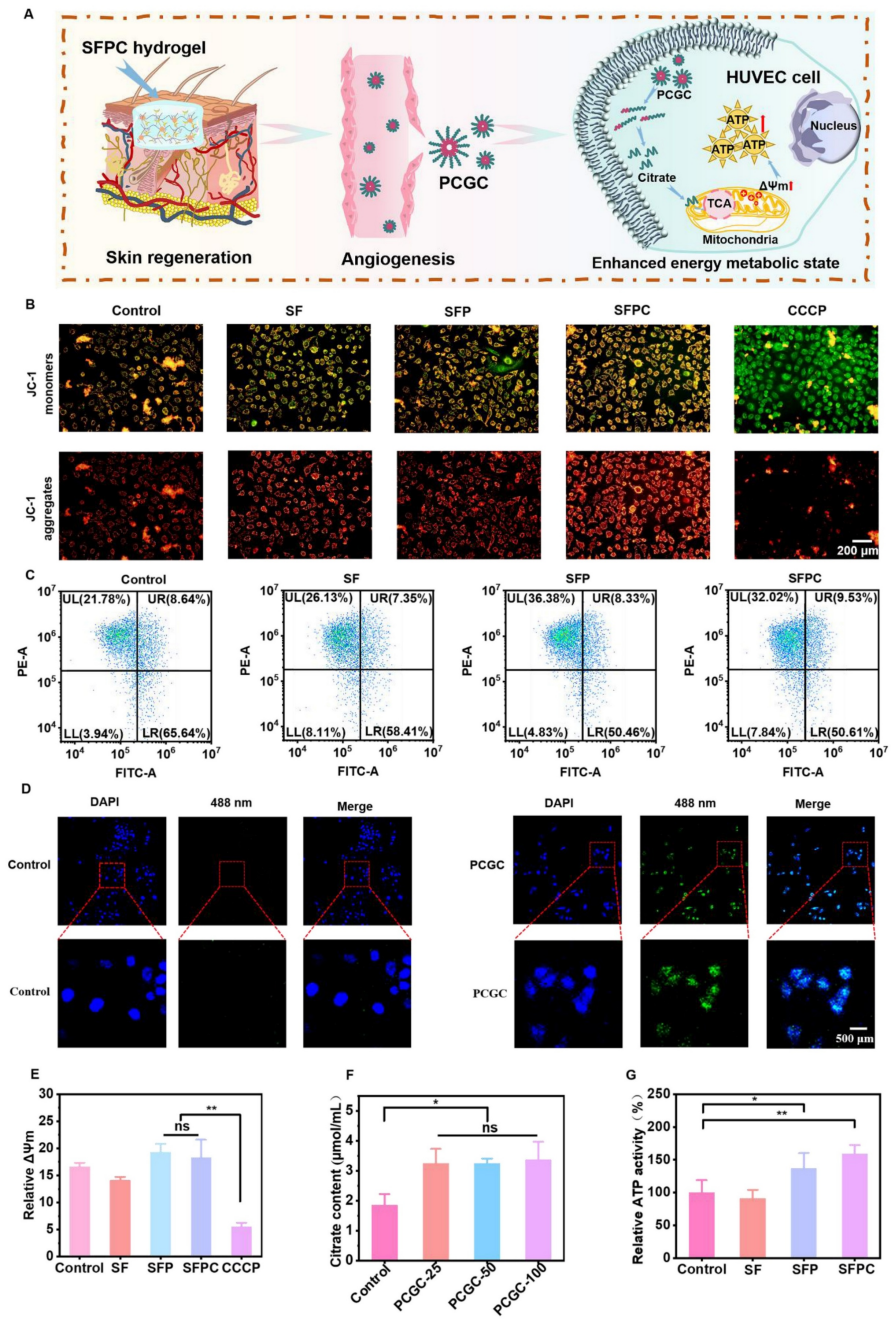
** SFPC hydrogels enhance cellular energy metabolism *in vitro* by promoting TCA cycle.** (A) Schematic diagram of SFPC hydrogel enhanced energy metabolism state of HUVEC cells. (B) JC-1 staining of HUVEC cells incubated with SFPC hydrogels. (C) Flow cytometry results of HUVEC cells after co-incubation with SFPC hydrogels for 48 h. (D) CLSM images of HUVEC cells after 6 h of incubation with PCGC in DEME medium at 37 °C, together with DAPI to stain the nuclei (blue). (E) The fluorescence intensity of JC-1 showed relevant mitochondrial membrane potential. (F) Quantitative statistics of citric acid uptake by HUVEC cells. (G) Relative intracellular ATP content after HUVEC cells incubated with SFPC hydrogels. ^**^p < 0.01, ^*^p < 0.05, ns means not significant.

Energy metabolism and tissue regeneration are closely linked. By enhancing energy metabolism, the production of anabolites is promoted, which accelerates mitosis and ultimately facilitates tissue repair 34. **Figure 5** shows the results of SFPC hydrogels in cellular bioenergetics in HUVEC cells. **Figure 5A** shows a schematic diagram of SFPC hydrogel enhanced energy metabolism state of HUVEC cells. The mitochondrial membrane potential (ΔΨm) is a key driver of ATP production in cells. A reduction in ΔΨm is commonly regarded as an early indicator of apoptosis. To assess mitochondrial membrane potential, JC-1 staining was conducted. The results showed a higher proportion of red JC-1 aggregates in the SFPC group compared to the control group, indicating elevated mitochondrial activity and enhanced cellular energy metabolism. In contrast, cells treated with CCCP, an oxidative phosphorylation inhibitor, exhibited predominant green JC-1 monomers under the microscope, reflecting a loss of mitochondrial membrane potential and the initiation of apoptosis (**Figure 5B**). Subsequently, flow cytometry was performed for cell profiling, and the results were consistent with the JC-1 staining analysis. JC-1 monomers detected in the FITC channel indicate reduced mitochondrial membrane potential, while JC-1 aggregates detected in the PE channel correspond to elevated potential. Cells located in the upper-left quadrant of the dot plot, characterized by strong red fluorescence and weak green fluorescence, represent a population with high mitochondrial membrane potential. The proportion of activated cells in the SFPC group reached 32.02%, significantly higher than that in the control group (21.78%), indicating that SFPC treatment enhanced mitochondrial membrane potential. Moreover, although the SFP group showed an activation rate of 36.38%, it was still lower than that of the SF group (26.13%). These results demonstrated that the incorporation of PCGC notably enhanced cellular metabolic activity (**Figure 5C**). As shown in **Figure 5E**, the introduction of PCGC significantly increased mitochondrial membrane potential. Notably, the cellular uptake of PCGC is a key prerequisite for SFPC hydrogels to enhance cellular metabolic activity. Due to its amphiphilic structure, PCGC can self-assemble into nanomicelles in physiological environments, facilitating its cellular internalization. Once internalized, PCGC can directly exert its bioactive effects within the cells (**Figure S2**). Subsequently, we observed PCGC uptake by HUVEC cells using CLSM. As shown in **Figure 5D**, a significant green fluorescence signal can be observed in the PCGC group, which was supposed to be the fluorescence emitted by PCGC itself. We evaluated the fluorescence properties of PCGC. When excited at 343 nm, PCGC exhibited an emission peak near 530 nm. Furthermore, as the PCGC concentration decreased, the fluorescence intensity diminished (**Figure S3**). The introduction of curcumin in PCGC, with its large delocalized π-conjugated system, β-diketone structure, and keto-enol tautomerism, together endow it with good fluorescence properties.

In contrast, no fluorescence was observed in the control group, indicating that PCGC was successfully internalized by HUVECs after 8 hours of incubation. To further investigate the impact of PCGC on the tricarboxylic acid (TCA) cycle, we measured intracellular citrate levels-a key intermediate metabolite-using a Citrate Microplate Assay Kit. The results showed that citrate levels in the PCGC-treated group were significantly higher than those in the control group, suggesting that PCGC enhances TCA cycle activity (**Figure 5F**). These results indicated that PCGC was effectively internalized by cells, leading to an increase in intracellular citrate levels. As a direct energy source, ATP plays a critical role in cellular metabolism by supporting the rapid synthesis of anabolic intermediates necessary for tissue repair and regeneration. To evaluate the bioenergetic effect of SFPC hydrogels, intracellular ATP levels were measured using an ATP Assay Kit. The results revealed that ATP production in the SFPC group was approximately 1.59 times higher than that in the control group, confirming the ATP-enhancing capability of SFPC. Furthermore, the introduction of PCGC significantly boosted energy output, with ATP production in the SFP group reaching 137.1% relative to the SF group (91.1%) (**Figure 5G**). In conclusion, these cellular energy metabolism studies demonstrated that SFPC hydrogels can degrade into PCGC, which is readily internalized by cells, leading to elevated intracellular citrate levels and enhanced mitochondrial membrane potential (ΔΨm) *via* participation in the TCA cycle. Collectively, these findings highlight the promising potential of SFPC hydrogels to accelerate tissue repair by modulating cellular energy metabolism.

### *In vivo* MRSA-infected wound repair evaluation

**Figure 6:**
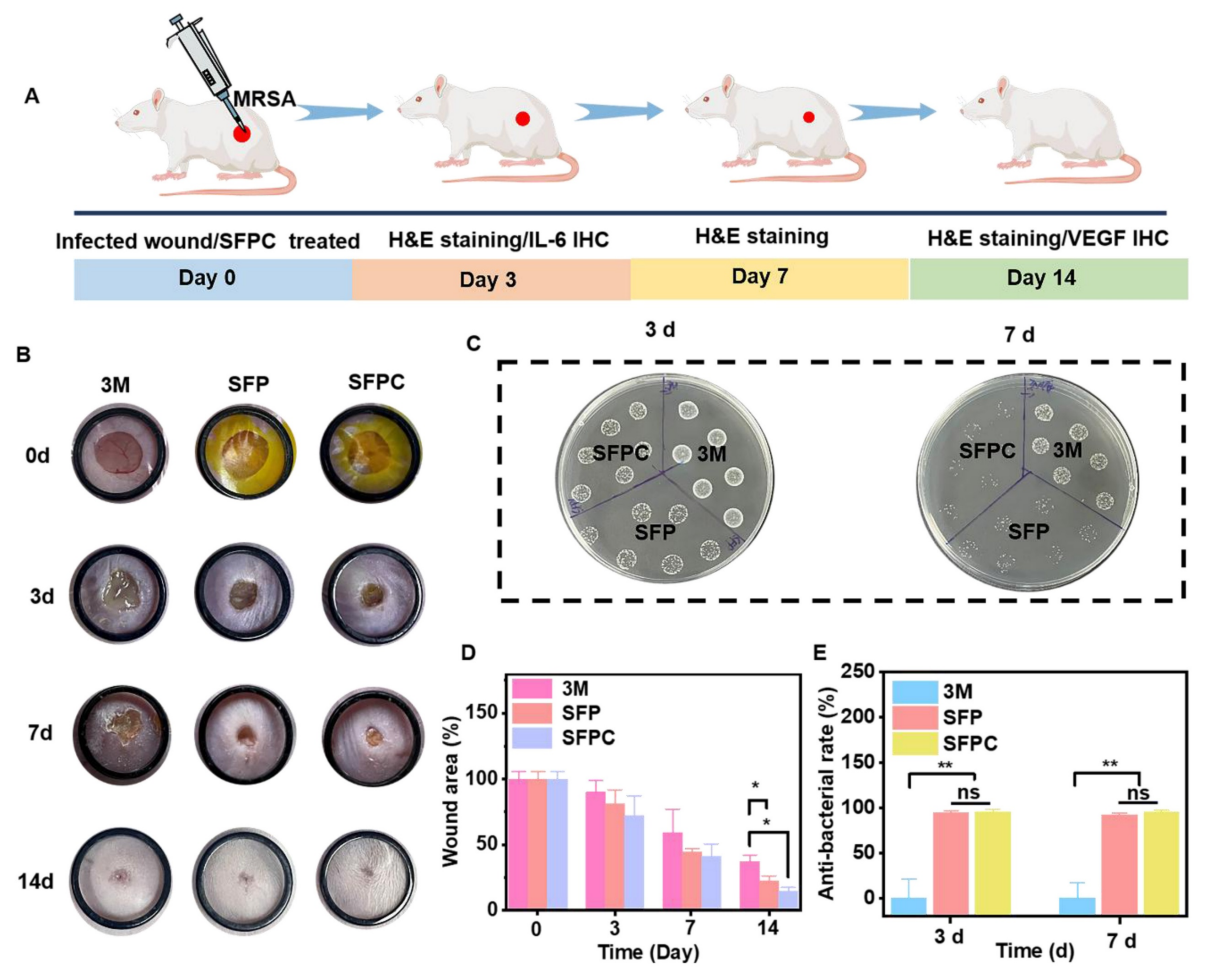
** Evaluation of *in vivo* anti-infection, wound healing and skin regeneration in MRSA infected mouse wound model.** (A) Schematic diagram of preparation and treatment of MRSA-infected mice. (B) Representative skin wound photographs treated with 3M, SFP hydrogel and SFPC hydrogel for 14 days. (C) MRSA images of different sample treatments *in vivo***.** (D) Quantification of the relative wound area of wounds. (E) Antibacterial ratio of various samples. ^**^p < 0.01, ^*^p < 0.05, ns means not significant.

**Figure 7:**
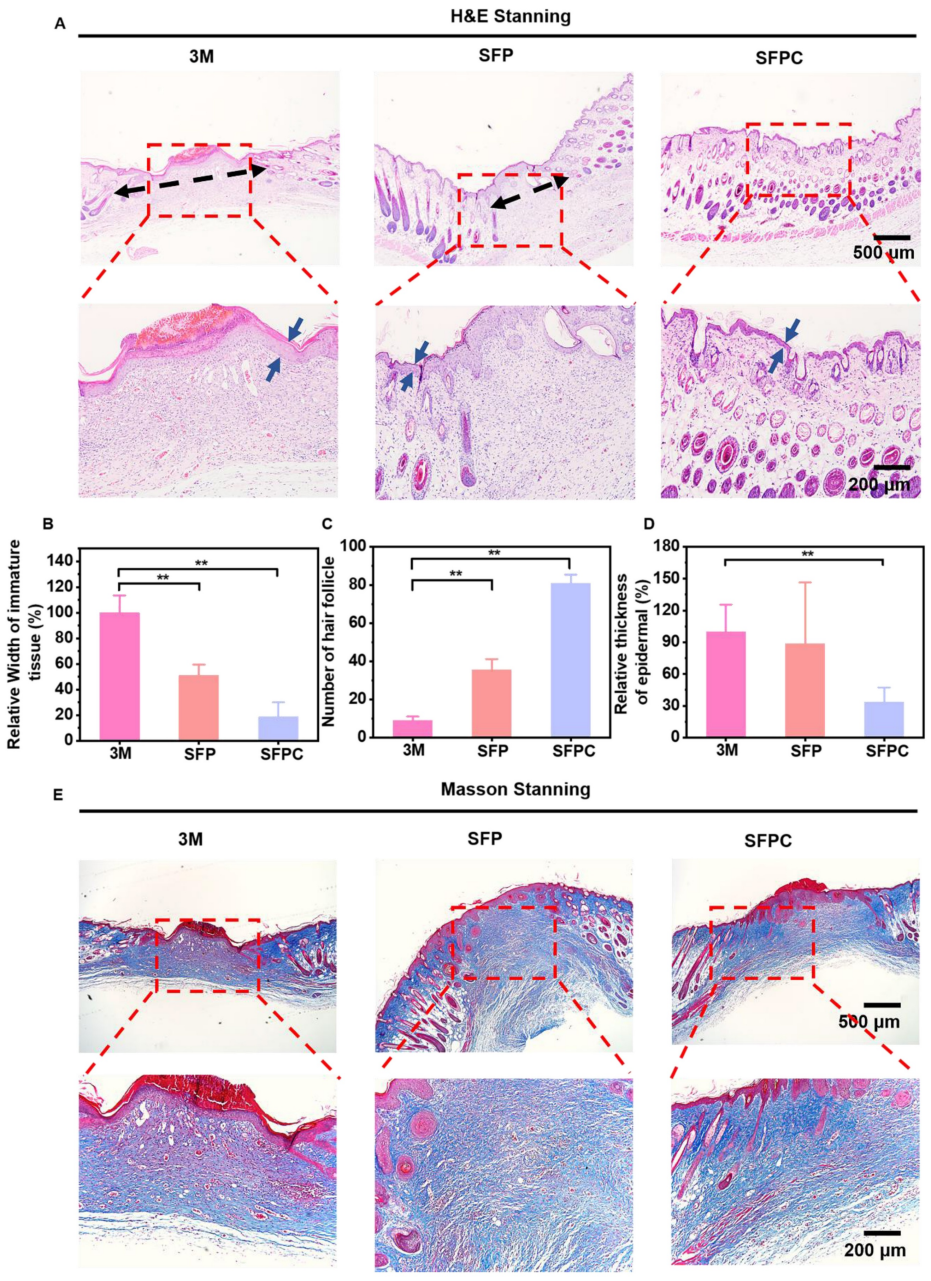
*** In vivo* wound healing and skin regeneration evaluation in MRSA infected mice wound model.** (A) Images of H&E-stained wound tissue sections after treated with various samples on day 14. Quantitative statistics of relative width of immature tissue (B), number of hair follicle (C), relative thickness of epidermal (D). *p < 0.05. **p < 0.01. (ns means not significant). (E) Images of masson stained wound tissue sections after treated with various samples on day 14. ^**^p < 0.01.

The effects of SFPC hydrogel on trauma repair were evaluated by a whole cortex injury model in MRSA-infected mice. **Figure 6** shows the results of the evaluation of SFPC hydrogel for MRSA infected wound repair. **Figure 6A** is a schematic diagram of the preparation and treatment of MRSA infected mice. **Figure 6B** shows the macroscopic healing progress of 3M, SFP hydrogel, and SFPC hydrogel at 3, 7, and 14 days of wound repair. On day 3, the inflammatory exudates, pus, *etc*. were visible on the trauma in 3M group. On the contrary, the SFP and SFPC groups had started to show crusting on the wounds, which proved the great advantage of antibacterial and anti-inflammatory of SFPC hydrogel. On day 14, the wounds in the SFPC group were substantially closed, while the 3M group still had a certain area of trauma present. Quantitative statistics had a consistent result, with the 14-day SFPC group having a significantly smaller wound area (14.4%) than the 3M group (36.9%) (**Figure 6D**). In addition, the *in vivo* antimicrobial results of the SFPC group were consistent with their *in vitro* antimicrobial results. On day 3, the results showed that the colony growth of SFPC group was significantly less than that of 3M group. On day 7, only a negligible number of colonies were visible in the SFP and SFPC, however, the 3M group still had a lot of colonies present (**Figure 6C**). The antimicrobial resistance of SFP and SFPC can reach almost 99.9% at both 3 and 7 days (**Figure 6E**). In addition, H&E staining was performed for further evaluation of the wound repair process. Compared with 3M, the SFPC group had completely healed wounds, as well as having more hair follicles and skin appendages present (**Figure 7A**). On day 14, the 3M group still exhibited some trauma, compared to the SFP group and the SFPC group, which displayed complete epidermal coverage and had significantly less immature tissue width than the 3M group (**Figure 7B**). Further, quantitative statistics of the number of hair follicles showed that the number of hair follicles in the SFP and SFPC groups was approximately 3.9 and 9.0 times that of the 3M group, respectively (**Figure 7C**). In addition, the wound tissues in the SFP group and SFPC treatment exhibited thinner epidermal thickness, which demonstrated accelerated wound healing as well as a satisfactory state of repair (**Figure 7D**). Subsequently, the wounds on day 14 were stained with Masson stain to observe the results of collagen deposition. As shown in **Figure 7E**, the density of collagen deposition in the SFPC group was significantly higher than that in the 3M group.

**Figure 8:**
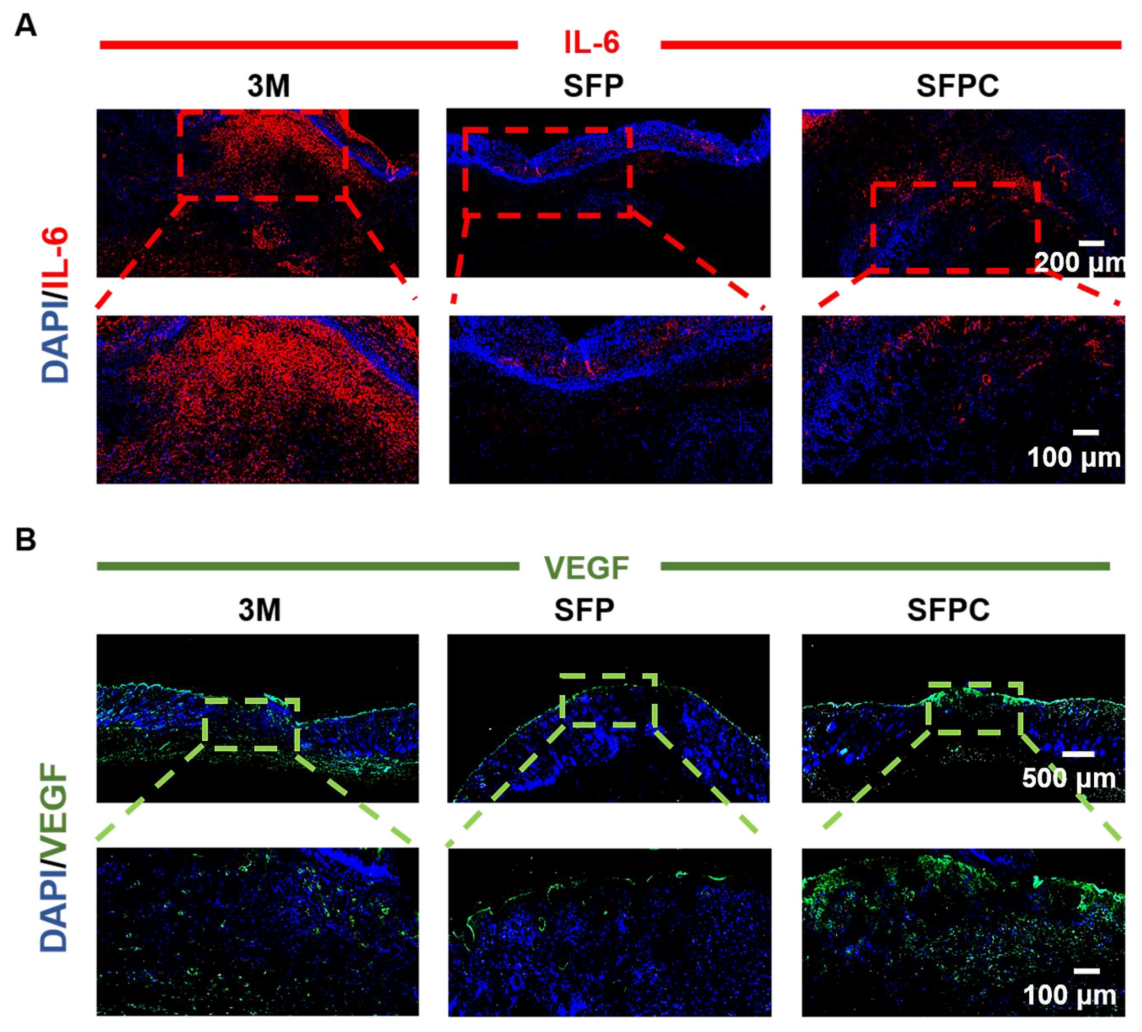
** Immunofluorescent staining assessment of wound healing *in vivo*.** (A) Representative IL-6 immunofluorescence staining of wound tissues on day 3. (B) Representative VEGF immunofluorescence staining of wound tissues on day 14.

To further reveal the *in vivo* anti-inflammatory effects of SFPC hydrogel, we evaluated the expression of the anti-inflammatory factor IL-6 in skin tissues on day 3. As shown in** Figure 8A**, the 3M group was characterized by more IL-6 positive areas, indicating that inflammation was still present. By contrast, IL-6 positive areas were significantly reduced in the SFP and SFPC groups due to favorable anti-inflammatory effects. The favorable anti-inflammatory effects of SFPC hydrogels are attributed to the properties of curcumin's own ROS scavenging and down-regulation of inflammatory factor expression levels. VEGF is regarded as a prominent marker of angiogenesis, and we evaluated the promotion of angiogenesis by SFPC hydrogels by immunohistochemical staining for VEGF 35. Positive signals in the trauma of the SFPC-treated group were significantly higher than those of the 3M group (**Figure 8B**). Taken together, these results indicated that SFPC hydrogel can effectively improve wound repair and regeneration of MRSA infections through antibacterial, anti-inflammatory, and promotion of vascularization.

## Discussion

In this study, we developed a multifunctional SFPC hydrogel *via* HRP/H_2_O_2_-mediated cross-linking, exhibiting self-healing capability, intrinsic fluorescence, excellent biocompatibility, broad-spectrum antibacterial activity, antioxidant and anti-inflammatory effects, ROS scavenging, and pro-vascularization properties. Notably, the hydrogel also enhanced cellular energy metabolism. In a murine model of MRSA-infected wounds, SFPC hydrogel significantly accelerated wound healing and tissue regeneration, demonstrating strong therapeutic potential.

This work highlights the multifaceted advantages of SFPC hydrogels in the treatment of MRSA-infected wounds and skin tissue remodeling. Firstly, the SFPC hydrogel exhibits a well-defined microporous structure that facilitates efficient oxygen and nutrient transport, thereby creating a favorable environment for cell proliferation and delivery. These properties underscore its potential applications in tissue engineering and regenerative medicine 36. Meanwhile, the SFPC hydrogel also exhibited excellent broad-spectrum antibacterial activity, which was primarily attributed to the inherent antimicrobial properties of Co^2+^ and the synergistic effects of curcumin and citric acid. The incorporation of curcumin further enhanced the hydrogel's anti-inflammatory performance by significantly downregulating the expression of pro-inflammatory cytokines, thereby accelerating the transition from the inflammatory phase to the proliferative phase during wound healing. In addition, the SFPC hydrogel promoted angiogenesis by facilitating endothelial cell migration and tube formation, along with the upregulation of angiogenic markers such as *VEGF* and *CD31*. Importantly, this study revealed a novel bioenergetic mechanism by which the poly citrate-based hydrogel contributed to tissue regeneration. The degradation products of SFPC hydrogel were internalized by cells, resulting in increased intracellular citrate levels. This citrate participated in the tricarboxylic acid (TCA) cycle, enhanced mitochondrial membrane potential, and boosted ATP production in endothelial cells. This elevated energy metabolic state played a critical role in supporting late-stage tissue repair and structural remodeling, offering a promising strategy for improving the healing of MRSA-infected wounds.

Compared with previously reported hydrogels for the treatment of MRSA-infected wounds, the SFPC hydrogel demonstrated several notable advantages. First, it was simple and efficient to prepare, with components derived from natural sources, ensuring excellent biocompatibility and laying a solid foundation for future clinical applications. Second, the inherent photoluminescent properties of the SFPC hydrogel enabled real-time fluorescence imaging, precise localization, visual tracking, and clarification of its therapeutic mechanism of action. Third, the SFPC hydrogel exhibited robust anti-inflammatory, antibacterial, and pro-angiogenic activities, which collectively facilitated the transformation of an inflammatory wound microenvironment into a regenerative one, highlighting its promising potential in tissue repair and regeneration. Fourth, beyond its multifunctional therapeutic effects, the SFPC hydrogel also promoted wound healing by participating in the cellular TCA cycle, enhancing ATP production, and accelerating cellular energy metabolism-thus providing a solid bioenergetic foundation for tissue regeneration at the cellular level. Finally, both PCGC and SF possessed reactive functional groups (e.g., hydroxyl, carboxyl, amino), which could be further modified and functionalized as needed to expand their biomedical applications. Taken together, these findings suggested that SFPC hydrogel represented a promising new candidate for use in disease treatment and regenerative medicine.

## Conclusions

In summary, we developed a multifunctional bioactive SFPC hydrogel with a unique capacity to enhance cellular energy metabolism, aiming to accelerate the repair and regeneration of MRSA-infected wounds. The SFPC hydrogel exhibited robust broad-spectrum antibacterial, anti-inflammatory, intracellular ROS-scavenging, and pro-angiogenic activities. Through Co^2+^ doping, the hydrogel stabilized the expression of *HIF-1α* and subsequently upregulated *VEGF* expression, thereby enhancing vascularization. Moreover, the SFPC hydrogel released PCGC, which was internalized by cells and degraded into citrate. This citrate entered the TCA cycle, increased the mitochondrial membrane potential, accelerated ATP synthesis, and ultimately promoted tissue repair and regeneration. As a multifunctional and bioactive wound dressing, the SFPC hydrogel demonstrated significant potential for clinical translation in the treatment of MRSA-infected wounds.

## Supplementary Material

Supplementary experimental section and figures.
