# Supplementary material for: Bioactive metal-protein matrix for promoting MRSA infection wound therapy through bioenergy-induced angiogenesis
Source: Theranostics. 2025 Jun 9;15(14):6882–900. doi: 10.7150/thno.112678 (PMC12203806; doi:10.7150/thno.112678)
Supplement: Supplementary file 1 — Supplementary experimental section and figures. [file thnov15p6882s1.pdf]

Supporting information

Bioactive Metal-Protein Matrix for Promoting MRSA Infection Wound Therapy through Bioenergy-Induced Angiogenesis

Sihua Li [a], Junping Ma [a], Liuyang Zhang [a], Xiaoyan Qu [a], Long Zhang [c], Qian Huang [a], Bo Lei [a, b, c]*

[a] *Key Laboratory of Shaanxi Province for Craniofacial Precision Medicine Research, College of Stomatology, Xi'an Jiaotong University, Xi'an 710000, China*

[b] *Frontier Institute of Science and Technology, Xi'an Jiaotong University, Xi'an 710000, China*

[c] *Department of Respiratory and Critical Care Medicine, The Second Affiliated Hospital of Xi'an Jiaotong University, Xi'an, 710004, China*

* To whom correspondence should be directed

E-mail: rayboo@xjtu.edu.cn

Experimental section

1.1 Hemocompatibility

The hemocompatibility of SFPC hydrogel was characterized by evaluating the hemolysis rate. Specifically, 2 mL of fresh mice blood was added into 50 mL phosphate buffered saline (PBS) and wash the blood 5-6 times. And then 500 μL SF, SFP, SFPC hydrogels and Triton X-100 were added into a 24-well plate containing 1 mL of treated blood. PBS and Triton X-100 were considered as the negative and positive controls, respectively. Subsequently, the 24-well plates were incubated for 1 h at 37 °C. Then 1 mL liquid were collected into tubes and all tubes were centrifuged at 1200 rpm for 20 min. Finally, a camera was used to take pictures of all tubes, the absorbance of the supernatant was determined at 540 nm by the SPECTROMAX I3, and the morphology of red blood cells were observed by the microscope. The hemolysis ratio was calculated as follows: Hemolysis (%) = $[(OD_h - OD_{PBS}) / (OD_{Triton\ X\text{-}100} - OD_{PBS})] \times 100$ %, where OD_h, OD_{PBS} and $OD_{Triton\ X\text{-}100}$ are the absorbance values of the samples, negative control (PBS) and positive control (Triton X-

100), respectively.

1.2 Antibacterial performance investigation

The antibacterial ability of the hydrogel is crucial for preventing bacterial infection in the wound area. Therefore, this study investigated the broad-spectrum antibacterial activity of SFPC hydrogel against Escherichia coli (*E. coli)*, staphylococcus aureus *(S. aureus)*, and methicillin-resistant staphylococcus aureus (MRSA). In particular, 300 μL of hydrogel containing different cobalt ions doping concentrations (0%, 1%, 2%) were added to the bottom of a 24-well cell culture plate, with an equal volume of Phosphate Buffered Saline (PBS) as the control group. Then, 10 μL of bacterial solution containing 10^6 CFU/mL was dropped onto the surface of the hydrogel, and incubated in a 37 °C incubator for a total of 2 h. After incubation, 990 μL of PBS was added to the hydrogel group and mixed thoroughly to collect the bacterial solution, while the control group received 990 μL of PBS. Subsequently, 10 μL of diluted bacterial solution (10^3 CFU/mL) was taken for further analysis. The antimicrobial rate of the hydrogel was calculated by the formula: Antimicrobial rate = $[1 - (N_{PBS} - N_{Sample})/N_{PBS}] \times 100\%$.

1.3 Anti-inflammatory evaluation

The Anti-inflammatory ability of SFPC hydrogel was evaluated by analyzing the expression of inflammatory factors (TNF-α, IL-1β) in the macrophages. Briefly, RAW 264.7 cells were plated in a 12 well plates and cultured in the Dulbecco's modified eagle medium (DMEM) with 10% fetal bovine serum (FBS) at 37 °C in an incubator with 5% CO_2. After cell adhesion, 100 ng/mL lipopolysaccharide (LPS) was added into 12 well plates to induce the macrophage M1 polarization for 12 h. The group without LPS was used as a control group. And then after 2 days of incubation with samples with different concentrations, Total cellular RNA was extracted by Trizol. Reverse transcription by the Reverse Transcription System (Roche) was then performed. The quantitative real-time polymerase chain reaction (qRT-PCR) was performed on the Applied Biosystems 7500 Fast Real-time PCR system and iTaq universal SYBR Green supermix. The ΔΔCt method

was used for gene expression analysis. β-Actin was used as a reference gene.

1.3 Vascularization analysis

The vascularization ability of SFPC hydrogel was evaluated by analyzing the expression of inflammatory factors (*VEGF*, *CD31*, and *HIF-1α*) in the macrophages. Briefly, HUVEC cells were plated in a 12 well plates and cultured in the Dulbecco's modified eagle medium (DMEM) with 10% fetal bovine serum (FBS) at 37 °C in an incubator with 5% CO_2. And then after 2 days of incubation with samples with different samples, Total cellular RNA was extracted by Trizol. Reverse transcription by the Reverse Transcription System (Roche) was then performed. The quantitative real-time polymerase chain reaction (qRT-PCR) was performed on the Applied Biosystems 7500 Fast Real-time PCR system and iTaq universal SYBR Green supermix. The ΔΔCt method was used for gene expression analysis. β-Actin was used as a reference gene.

Figures

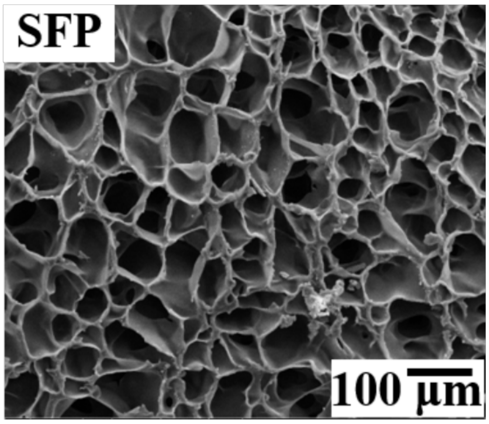

Figure S1. Typical SEM images of SFP hydrogeel.

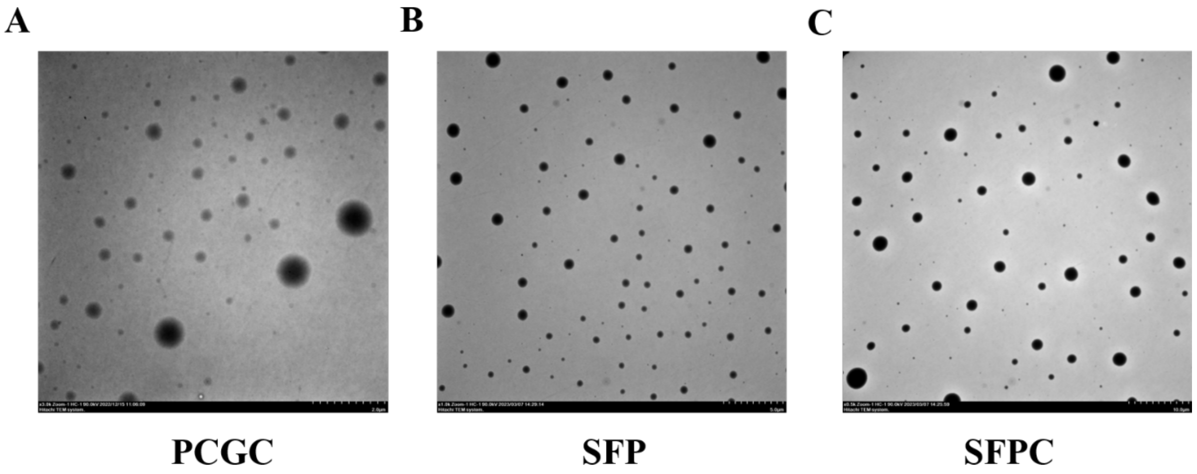

Figure S2. Typical TEM images of PCGC(A), SFP(B) and SFPC(C).

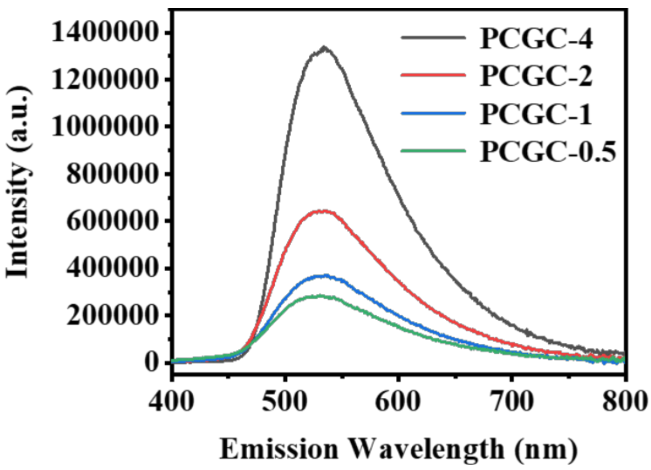

Figure S3. Emission spectra of different concentrations of PCGC at 343 nm excitation wavelength.
